# Analysis of Cytogenetic Abnormalities in Iranian Patients with Syndromic Autism Spectrum Disorder: A Case Series

**DOI:** 10.22037/ijcn.v16i4.34843

**Published:** 2022-03-14

**Authors:** Mohammad Reza GHASEMI, Peyman ZARGARI, Hossein SADEGHI, Saman BAGHERI, Behnia SADEGHGI, Reza MIRFAKHRAIE, Mahdis EKRAMI, Sepideh MOHAMMADI SARVALEH, Farzad HASHEMI GORJI, Katayoon RAZJOUYAN, Davood OMRANI, Hyung goo KIM, Mohammad MIRYOUNESI

**Affiliations:** 1 Department of Medical Genetics, Faculty of Medicine, Shahid Beheshti University of Medical Sciences, Tehran, Iran `; 2 Genomic Research Center, Shahid Beheshti University of Medical Sciences, Tehran, Iran.; 3 Center for Comprehensive Genetic Services, Shahid Beheshti University of Medical Sciences, Tehran, Iran.; 4 Psychiatric department, Shahid Beheshti University of Medical Sciences, Tehran, Iran.; 5 Neurological Disorders Research Center, Qatar Biomedical Research Institute, Hamad Bin Khalifa University, Doha, Qatar.

**Keywords:** Autism spectrum disorder, Syndromic, Karyotype, Array-Comparative genomic hybridization

## Abstract

**Objective:**

Autism spectrum disorder (ASD) is a heterogeneous neuropsychiatric group of pervasive developmental disorders mainly diagnosed through the complex behavioral phenotype. According to strong genetic involvement, detecting the chromosome regions and the key genes linked to autism can help to elucidate its etiology. The present study aimed to investigate the value of cytogenetic analysis in syndromic autism and find an association between autism and chromosome abnormalities.

**Materials & Methods:**

Thirty-six autistic patients from 30 families were recruited, clinically diagnosed with the Diagnostic and Statistical Manual of Mental Disorders (5th ed.; DSM-5). The syndromic patients with additional clinical features (including development delay, attention deficit, hyperactivity disorder, seizure, and language and intellectual impairment) were selected due to elevating the detection rate. Cytogenetics analysis was performed using GTG banding on the patients’ cultured fibroblasts. Moreover, array-comparative genomic hybridization (CGH) was also performed for patients with a *de novo* and novel variant.

**Results:**

Karyotype analysis in 36 syndromic autistic patients detected chromosomal abnormalities in 2 (5.6%) families, including 46,XY,dup(15)(q11.1q11.2) and 46,XX,ins(7)(q11.1q21.3)dn. In the latter, array-CGH detected 3 abnormalities on chromosome 7, including deletion and insertion on both arms: 46,XX,del(7)(q21.11q21.3),dup(7)(p11.2p14.1p12.3)dn.

**Conclusion:**

We reported a novel and *de novo* cytogenetic abnormality on chromosome 7 in an Iranian patient diagnosed with syndromic autism. However, the detection rate in syndromic autism was low, implying that it cannot be utilized as the only diagnostic procedure.

## Introduction

Autism spectrum disorder (ASD) is a group of childhood neurological and developmental disabilities described by the triad of abnormal social interest, communication deficit, and restricted/repetitive behavior with a strong hereditary basis (1, 2). Because of the wide range of severity and types, currently, it is assigned as a spectrum disorder, not only clinically but also genetically.

Depending on the presence or absence of several forms of dysmorphic features and neurobehavioral manifestations, ASD is categorized as a syndromic or non-syndromic (idiopathic) disorder, respectively. The causes of this major public health problem are still mostly unknown; however, there may be multiple types of causes, mostly comprising environmental and genetic causes (3). Based on twin studies using a quantitative meta-analysis approach, the heritability of ASD is estimated between 64% and 91%, implying a strong genetic effect (4). 

Syndromic autism is characterized as one of the neurodevelopmental disorders, accompanying further medical aspects that are frequently associated with chromosomal abnormalities. There is convincing evidence of a principally genetic role in autism and insignificant shared environmental impacts; however, the etiology remains largely unexplained in most cases (4). 

From the genetic point of view, ASD is highly heterogeneous and frequently related to chromosomal abnormalities as it is detectable in some syndromes such as fragile X syndrome and tuberous sclerosis (5-7). Microscopically noticeable chromosomal modifications have been explained in about 5% of cases (8). Almost every chromosome has been proved to be linked to autism (9). Some chromosomal aberrations have been frequently reported; the most frequent abnormalities are 15q11–q13 duplications, as well as 2q37, 22q11.2, and 22q13.3 deletions (10). Common chromosomal imbalances associated with ASD locate on chromosomes 1, 7, 15, 16, 17, and 22 (11). 

In the current study, our goal was to estimate the diagnostic productivity of cytogenetic techniques (karyotype) in syndromic autism. 

## Materials & Methods

The current study recruited 36 patients from 30 families with at least one of the children having ASD confirmed by the diagnostic criteria of ASD based on the Diagnostic and Statistical Manual of Mental Disorders (5th ed.; DSM-5) (2). These families were referred to the Medical Genetic Clinic of Center for Comprehensive Genetic Services (CCGS), Shahid Beheshti University of Medical Sciences (SBMU) from the psychiatric department, Imam Hossein Hospital. They were 30 males and 6 females (male/female ratio: 5/1) ranging from 3 to 17 years old. Complete clinical assessments and pedigree information were characterized before any laboratory performance. The pedigrees of the families were drawn in detail, including sex, age, consanguinity, and any background or similar conditions in the family for each case. To find the probable chromosomal abnormalities, the probands of the families were cytogenetically analyzed. 

Initial tests to exclude genetic syndromes were based on phenotypes. Subsequently, peripheral blood lymphocytes were analyzed using a high-resolution GTG-banding technique according to the laboratory standard protocol with a resolution of 350-400 bands (12). At least 20 metaphases were analyzed for each case.

To estimate the accurate size of the abnormal region, whole-genome Oligo-Array comparative genomic hybridization (CGH) was carried out using SurePrint G3 ISCA V2 8X60K whole-genome Oligo-Array version 2 according to the manufacturer protocol. The array consists of 60,000 spots with overall median probe spacing of at least 60 Kb that is detected close to 500 targeted disease regions. The sample was hybridized against the female sample (6151) used as a reference.

Written informed consent was signed by the parents on behalf of the participants before any accomplishment. The Ethics Committee of Shahid Beheshti University of Medical Sciences, Tehran, Iran, approved this study (code: IR.SBMU.MSP.REC.1399.472). 

## Results

Thirty-six Iranian patients with syndromic ASD, including 30 males and 6 females, from Imam Hossein Hospital, met the diagnostic criteria of autistic disorder and karyotyping analysis. All general and clinical data about these cases are available in Table 1. 

Patients’ electroencephalography (EEG) and magnetic resonance imaging (MRI) scans showed no abnormalities. Eight (22.22%) families were consanguineous. Twenty-one pregnancies were normal vaginal deliveries compared to 13 caesarian sections (2 pregnancies were twins). Thirteen (36.1%) patients were characterized by developmental delay, and 26 (87%) had language impairment. Although only 6 (16.6%) patients had at least a seizure during their life, 25 (69.4%) participants have attention deficit hyperactivity disorder (ADHD). 

Two (5.5%) patients had chromosomal abnormalities. Details about their clinical and cytogenetic information are as follows: 

**Case 1**

**Figure 1:**
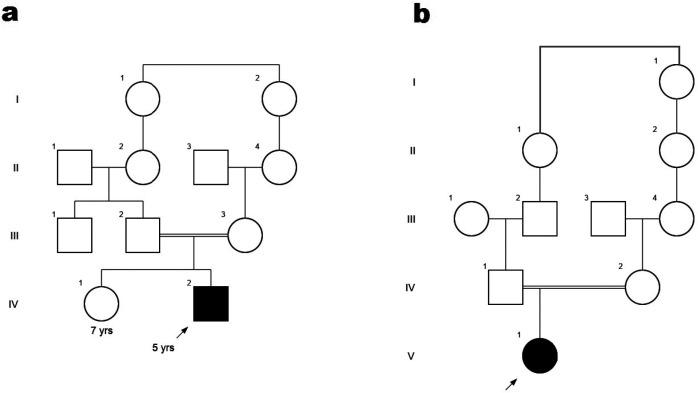
Pedigrees of the families with chromosome abnormalities; solid squares and circles illustrate affected individuals with ASD. (A) IV-2 is a proband as a case 1. (B) V-1 is a proband as a case 2

This case was a 5-year-old boy, the second child of healthy parents with consanguineous marriage (Figure 1). He was born by normal vaginal delivery after an uncomplicated pregnancy with normal birth parameters.

Parents were phenotypically normal. Behavioral anomalies (including speech delay, intellectual disability, repetitive behavior, and visual impairment) were diagnosed. Neither seizure nor ADHD was observed.

EEG and cerebral MRI showed no anomalies. Thus, ASD level 1 was suspected, and the diagnosis was confirmed using DSM-5 criteria by a pediatric psychiatrist.

**Figure 2:**
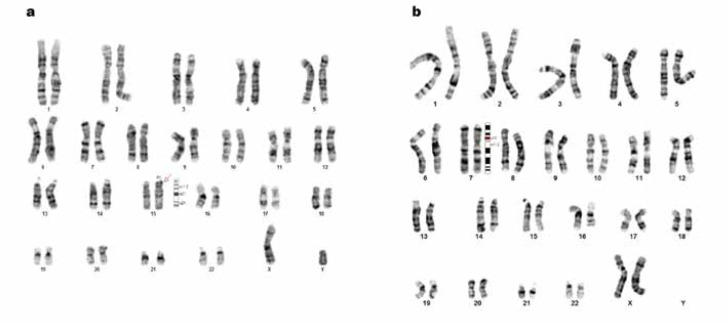
Abnormal results of cytogenetic (karyotype) analysis of cases 1 and 2. (A) Abnormal G-banded chromosome 15 was observed in all spreads; 46, XY, dup (15)(q11.1q11.2). (B) Abnormal G-banded chromosome 7 was observed in all spreads; the first karyotype result is 46, XX, ins(7)(q11.1q21.3)(dn). The normal ideograms of the revealed chromosome are according to the International System for Human Cytogenetic Nomenclature (2016)

Twenty metaphase spreads were analyzed, and interstitial duplication within the long arm of chromosome 15 from bands q11.1 to q11.2 was observed in all metaphases (Figure 2). 

**Case 2**

This case, a 9-year-old girl, is the only child of consanguineous and healthy parents born by cesarean delivery. Mother had a normal pregnancy. Ultrasonic examinations did not show any abnormality. 

The patient has a speech delay. Her EEG and MRI were completely normal, and she had no seizure. She has a mild intellectual disability and ADHD. 

**Figure 3:**
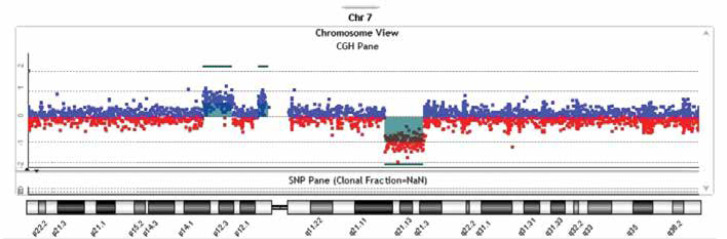
Array CGH profile of chromosome 7 using the SurePrint G3 ISCA V2 8X60K whole-genome Oligo-Array version 2

ASD was confirmed based on the DSM-5 criteria for autism by a pediatric psychiatrist. Chromosome analysis revealed abnormalities in all 20 studied metaphases, including an increase in the size of the long arm of chromosome 7 from bands q11.2 to q21.3 (Figure 2). In whole-genome Oligo-Array CGH (Figure 3), the following imbalances were detected:

- pathogenic gain of 7.04 Mb on 7p14.1p12.3 from nucleotides 41443439 to 48482470,
- pathogenic gain of 2.443 Mb on 7p11.2 from nucleotides 54549051 to 56992621, and
- pathogenic loss of 9.3 Mb on 7q21.11q21.3 from nucleotides 84592798 to 93891763.

## Discussion

In our investigation, 36 syndromic ASD patients were recruited from 30 Iranian families. Cytogenetic analysis revealed 2 chromosome abnormalities in 2 patients from different families (Figure 2). The first abnormality is a duplication within the long arm of chromosome 15 from q11.1 to q11.2, which is one of the most common cytogenetic aberrations in autistic patients. According to DSM-5, this patient had persistent deficits in all 3 areas of social communication and interaction. Genetically, studies display a chromosome duplication in 15q11.1-q11.2, which results in autism, epilepsy, ataxia, and developmental and language delays (13, 14). However, ataxia and epilepsy were not detected in our patient. For the first time, this duplication was reported in 1993 by Clayton-Smith in a patient with developmental delay and ataxia, similar to Angelman syndrome (15). In 1994, 3 patients with autistic disorder were reported to be associated with this duplication (13, 16). Two autistic patients in 2003 with normal EEG and MRI were reported, who had a 15q11-q13 inverted duplication (17). Subsequently, a study on 2 families demonstrated multigenerational maternal inheritance with this duplication; 4 out of 5 children have autistic features (18). Furthermore, a 33-year-old woman with poor motor skills and stereotyped movements associated with a microduplication in 15q11-q13 was reported in 2009 (19). 

This region contains a cluster of low-copy repeats located at breakpoints 1 and 2 (BP1, BP2). The BP1-BP2 region contains 4 conserved genes, including *NIPA1*, *NIPA2*, *CYFIP1*, and *TUBGCP5* (14). According to our results, the *CYFIP1* gene (which is located in the duplication region) has been duplicated in our patient. Cytoplasmic fragile X mental retardation 1 *FMR1* interacting protein 1 (*CYFIP1*) encodes a protein that regulates cytoskeletal dynamics and protein translation. This protein interacts with fragile X mental retardation protein (FMRP) and translation initiation factor 4E (eIF4E) to inhibit FMR1 translation in the brain, which is important in neuropsychiatric disorders (20). 

The first abnormality is duplication within the long arm of chromosome 15 from bands q11.1 to q11.2, which is one of the most common cytogenetic aberrations in autistic patients.

The second child with chromosome abnormality was first detected by the karyotype technique, including a duplication within the long arm of chromosome 7 from bands q11.1 to q21.3. She showed persistent deficits in all 3 areas of social communication and interaction based on DSM-5. The proband displayed a wide range of intellectual disabilities, speech delays, and ADHD.

Because of the critical region on chromosome 7, whole-genome Oligo-Array CGH was carried out using SurePrint G3 ISCA V2 8X60K whole-genome Oligo-Array version 2. According to genetic screening, the following imbalances were detected: arr[GRCh37] 7p14.1p12.3(41443439_48482470)x3, arr[GRCh37] 7p11.2(54549051_56992621)x3, and arr[GRCh37] 7q21.11q21.3(84592798_93891763)x1.

According to the American College of Medical Genetics and Genomics (ACMG) classification, the gains of 7.04 Mb and 2.443 Mb on 7p14.1p12.3 and 7p11.2, respectively, are pathogenic. Moreover, the loss of 9.3 Mb on 7q21.11q21.3 is also pathogenic (Figure 3);{46,XX,del(7)(q21.11q21.3),dup(7)(p11.2p14.1p12.3)dn}. These regions contain several OMIM genes, and all of these imbalances are considered pathogenic (Table 2).

This case with a pathogenic gain of 7.04 Mb on 7p14.1p12.3, insertion of 2.443 Mb on 7p11.2, and loss of 9.3 Mb on 7q21.11q21.3 was compatible with partial trisomy of 7p14.1p12.3 and 7p11.2, as well as partial monosomy of 7q21.11q21.3. According to our study, the 7q21.11q21.3 region was proposed as a susceptibility locus for neurodevelopmental disorders.

Despite our limitations (such as a small sample size), the detection rate of the karyotype in our subjects (36 syndromic autistic patients) is 2 out of 36 (5.5%), in line with another study (21). 

**Table 1:** General and clinical features of 36 cases from 30 families

**Other sign and symptoms**	**Intellectual statement**	**ADHD** ^+^	**Seizure**	**Language Condition**	**Development**	**D** ^#^	**Karyotype result**	C*	**Age**	**Sex**	**No**
Visual Impairment	Moderate	No	No	Speech delay	Delay	NVD	46, XY, dup(15)(q11.1q11.2)	Yes	7	M	1
-	Normal	Yes	No	Speech delay	Normal	CS	46, XX	No	6	F	2
Meconium aspiration syndrome (MAS), Congenital insensitivity to pain (CIP)	Normal	Yes	No	Speech delay	Normal	CS	46, XY	No	8	M	3
-	Moderate	Yes	No	Normal	Delay	NVD	46, XY	No	15	M	4
Learning Disability	Moderate	Yes	No	Speech delay	Normal	NVD	46, XY	Yes	13	M	5
Nuchal cord Hospitalized for jaundice	Normal	Yes	No	Speech delay	Normal	CS	46, XY, 16qh+	No	7	M	6
Severe Learning disability Myasthenia face First seizure: Heart failure for 2 minutes after Pneumonia	Normal	No	Yes	Dysarthria	Normal	CS	46, XX	Yes	10	F	7
Head trauma	Mild	Yes	No	Speech delay	Normal	NVD	46, XX	No	8	F	8
Learning disability	Moderate	Yes	No	Normal	Delay	CS	46, XY	Yes	15	M	9
Learning disability	Moderate	Yes	No	Normal	Delay	NVD	46, XY	No	17	M	10
Hospitalized for jaundice (Asperger Syndrome)	Normal	Yes	No	Speech delay	Normal	CS	46, XY	No	11	M	11
-	Mild	Yes	Yes	Normal	Delay	NVD	46, XY	No	16	M	12
-	Moderate	Yes	No	Speech delay	Normal	CS	46, XY	No	10	M	13
-	Mild	Yes	No	Normal	Delay	NVD	46, XY	Yes	10	M	14
-	Moderate	Yes	No	Speech delay	Normal	NVD	46, XY	No	2	M	15
-	Moderate	Yes	No	Speech delay	Normal	NVD	46, XY	No	3	M	16
Lymph node inflammation in the neck that resolves after surgery	Mild	No	No	Speech delay	Normal	NVD	46, XY	No	7	M	17
-	Mild	Yes	No	Speech delay	Normal	NVD	46, XX	No	8	F	18
-	Moderate	Yes	No	Speech delay	Normal	NVD	46, XY, 16qh+	No	6	M	19
-	Mild	No	No	Speech delay	Delay	NVD	46, XY	No	5	M	20
-	Moderate	No	No	Speech delay	Delay	NVD	46, XY	No	12	M	21
Learning Disability	Moderate	No	No	Speech delay	Delay	NVD	46, XY	No	17	M	22
Sleep problem Walking Problem	Moderate	No	No	Speech delay	Normal	NVD	46, XY	Yes	9	M	23
	Mild	Yes	No	Speech delay	Normal	CS	46, XX, ins(7)(q11.1q21.3)dn	Yes	9	F	24
delayed Necked and Delayed Head Control Strabismus	Moderate	Yes	No	speech Delay	Delay	CS	46, XY	No	9	M	25
delayed Necked and Delayed Head Control Strabismus	Moderate	Yes	Yes	speech Delay	Delay	CS	46, XY		8	M	
Restlessness at birth	Moderate	Yes	No	Speech delay	Delay	NVD	46, XY	No	9	M	26
Inguinal Hernia	Moderate	Yes	Yes	Normal	Delay	NVD	46, XY		8	M	
Twin Walking delay Strabismus	Moderate	Yes	No	Speech delay	Normal	CS	46, XY, 16qh+	No	3	M	27
							46, XY, 16qh+		3	M	
Twin Walking delay Normal HPLC for Aminoacid	Moderate	No	Yes	Speech delay	Normal	CS	46, XY	No	5	M	28
							46, XY		5	M	
Jaundice at birth	Moderate	Yes	No	Speech delay	Normal	NVD	46, XY	Yes	6	M	29
Jaundice at birth	Moderate	Yes	No	Speech delay	Normal	NVD	46, XY		4	M	
Mild microcephaly (like his father) Learning Disability	Moderate	Yes	Yes	Speech delay	Normal	CS	46, XY		13	M	30
Glaucoma at birth	Moderate	Yes	No	Speech delay	Normal	CS	46, XX		4	F	

* Consanguinity

^# ^Delivery type. NVD: Normal vaginal delivery; CS: caesarian section.

^+^ Attention deficit hyperactivity disorder (ADHD)

**Table 2:** Characterization of related regions for the chromosomal abnormalities detected in the case 2

**Gain of 7.04 Mb on 7p14.1p12.3**		
**MIM**	**Gene**	**Related phenotype**
165240	GLI3	GLI-KRUPPEL family member 3;
109750	BLVRA	biliverdin reductase A;
138079	GCK	glucokinase;
607707	CAMK2B	calcium/calmodulin-dependent protein kinase II-beta;
607929	CCM2	CCM2 gene
175700	GCPS	GREIG cephalopolysyndactyly syndrome;
146510	PHS	Pallister-hall syndrome;
174200	PAPA1	polydactyly, postaxial, type A1;
614156	HBLVD	hyperbiliverdinemia;
125853	T2D	type 2 diabetes mellitus;
617799	MRD54	mental retardation, autosomal dominant 54
603284	CCM2	cerebral cavernous malformations 2
		
**Gain of 2.443 Mb on 7p11.2**		
131550	EGFR	epidermal growth factor receptor
172480	PSPH	phosphoserine phosphatase
616244	XCHCH2	coiled-coil-helix-coiled-coil-helix domain-containing protein 2
211980	-	lung cancer
614023	PSPHD	phosphoserine phosphatase deficiency
616710	park22	Parkinson disease 22, autosomal dominant
		
**Loss of 9.3 Mb on 7q21.11q21.3**		
171060	ABCB4	ATP-binding cassette, subfamily b, member 4
171050	ABCB1	ATP-binding cassette, subfamily b, member 1
603709	ADAM22	a disintegrin and metalloproteinase domain 22
604001	AKAP9	a-kinase anchor protein 9
614972	ICP3	cholestasis, intrahepatic, of pregnancy 3
120080	-	colchicine resistance
617933	DEE61	developmental and epileptic encephalopathy 61
611820	LQT11	long QT syndrome 11
		

## In Conclusion

We concluded that the karyotype could help syndromic ASD patients to distinguish genetic causes as one of the first-tier tests––but not only. We also reported for the first time a novel and de novo chromosome abnormality on both arms of chromosome 7 in syndromic autistic patients, with the help of array CGH(46, XX,del(7)(q21.11q21.3),dup(7)(p11.2p14.1p12.3)dn

## Author Contributions

Completed recruitment and clinical confirmation performed by KR, M-RG, and MM. BS, ME, SM-S, M-RG, and MM performed laboratory experiments. M-RG, SB, and PZ wrote the manuscript. M-RG, FH-G, SB, HS, RM, DO, H-GK, and MM contributed to the revisions of the manuscript. The final revision has been confirmed and essential ideas into the revisions of the manuscript have been provided by all authors.

## Conflict of Interest

The authors declare no conflict of interest
